# Supplementary material for: The MRAP2 accessory protein directly interacts with melanocortin-3 receptor to enhance signaling
Source: bioRxiv. 2024 Nov 6:2024.11.06.622243. Preprint. [Version 1] doi: 10.1101/2024.11.06.622243 (PMC11580913; doi:10.1101/2024.11.06.622243)
Supplement: Supplement 1 [file media-1.pdf]

Supplementary Appendix

Figure S1 Expression of MRAP2 and MC3R in the human hypothalamus measured by spatial transcriptomics

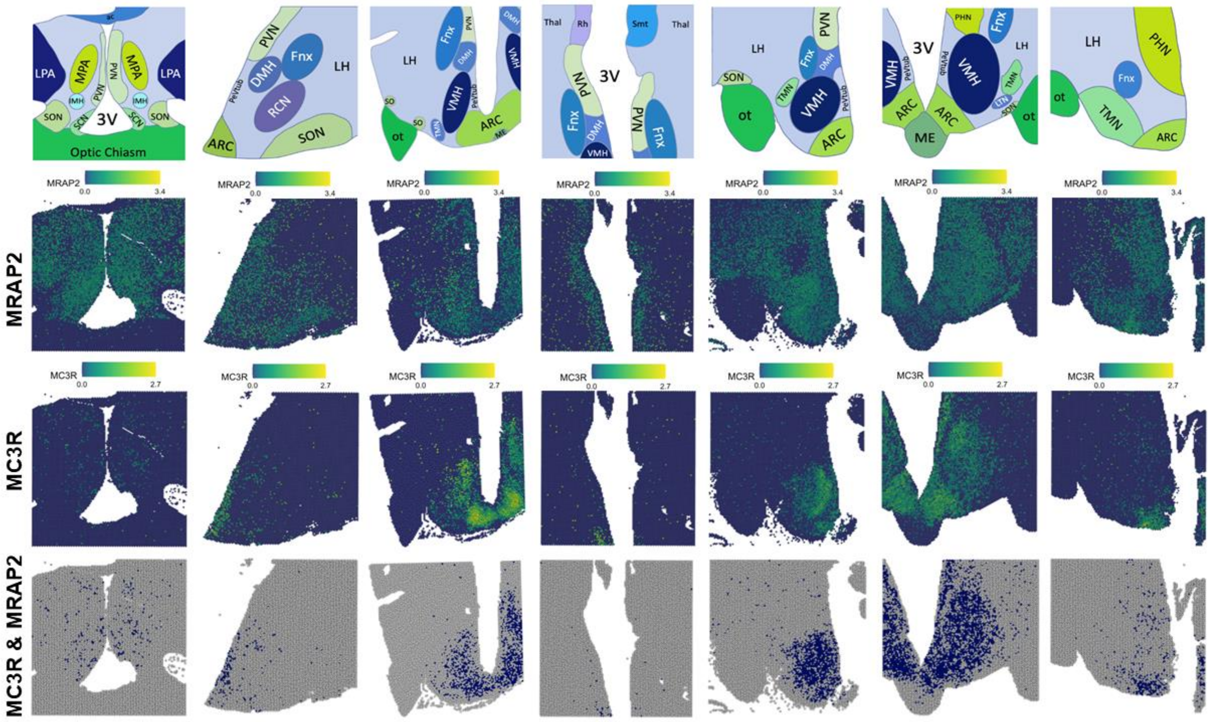

Spatial transcriptomic data showing log-normalized expression of MRAP2 and MC3R in the human hypothalamus. The cartoon shows the major regions of the hypothalamus (ARC, arcuate nucleus; DMH, dorsomedial hypothalamus; Fnx, fornix; LH, lateral hypothalamus, LTN, lateral tuberal nucleus; ME, medial eminence; MPA, medial preoptic area; PeVtub, periventricular nucleus; PHN, posterior hypothalamic nucleus; SCN, suprachiasmatic nucleus; SON, supraoptic nucleus; TMN, tuberomammillary nucleus, VMH, ventromedial nucleus of the hypothalamus), with areas in which MRAP2 and MC3R are co-expressed shown at the bottom (indicated in blue).

Figure S2 **MC3R plasmids express, traffic and signal normally**

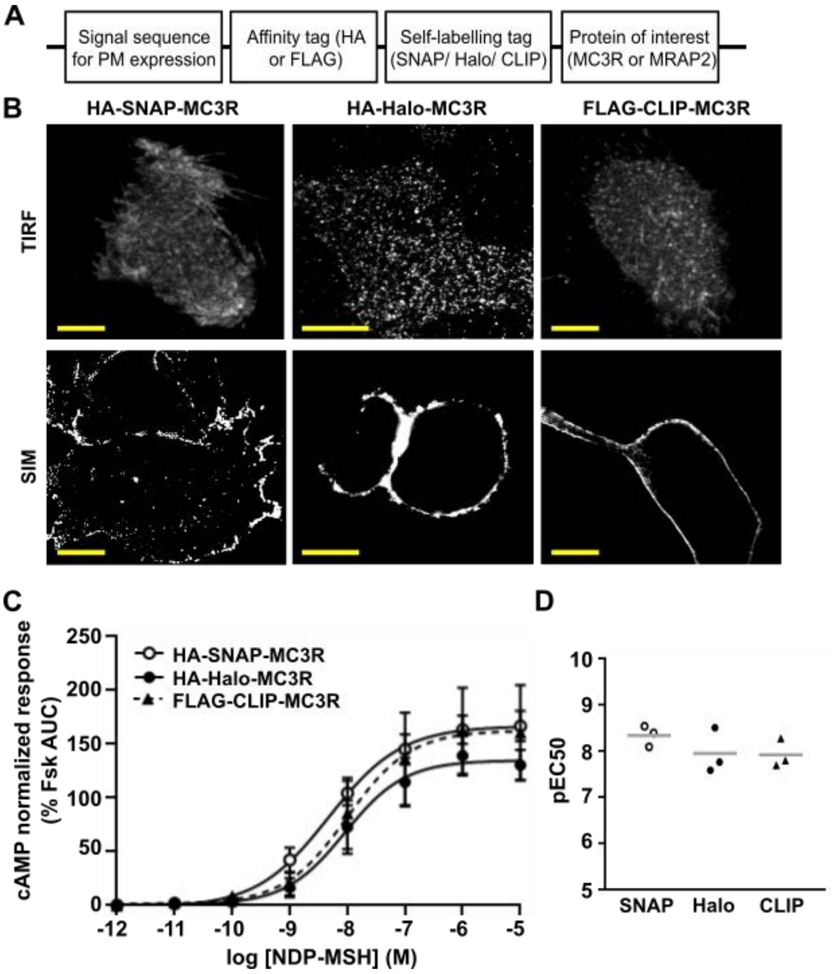

(**A**) Schematic showing the construction of the MC3R and MRAP2 plasmids with a signal sequence, affinity tag (HA or FLAG), self-labeling tag (SNAP, Halo or CLIP) and the protein of interest, (**B**) Imaging showing expression of the three MC3R constructs at the cell surface by TIRF and SIM. Scale, 5 µm. (**C**) MC3R-induced cAMP responses measured by Glosensor in cells transfected with the three MC3R plasmids and (**D**) pEC50. AUC was used to generate a dose-response and expressed relative to basal responses. N=3. Statistical analyses were performed by two-way ANOVA with Sidak's multiple-comparisons test.

Figure S3 MRAP2 plasmids express and signal normally

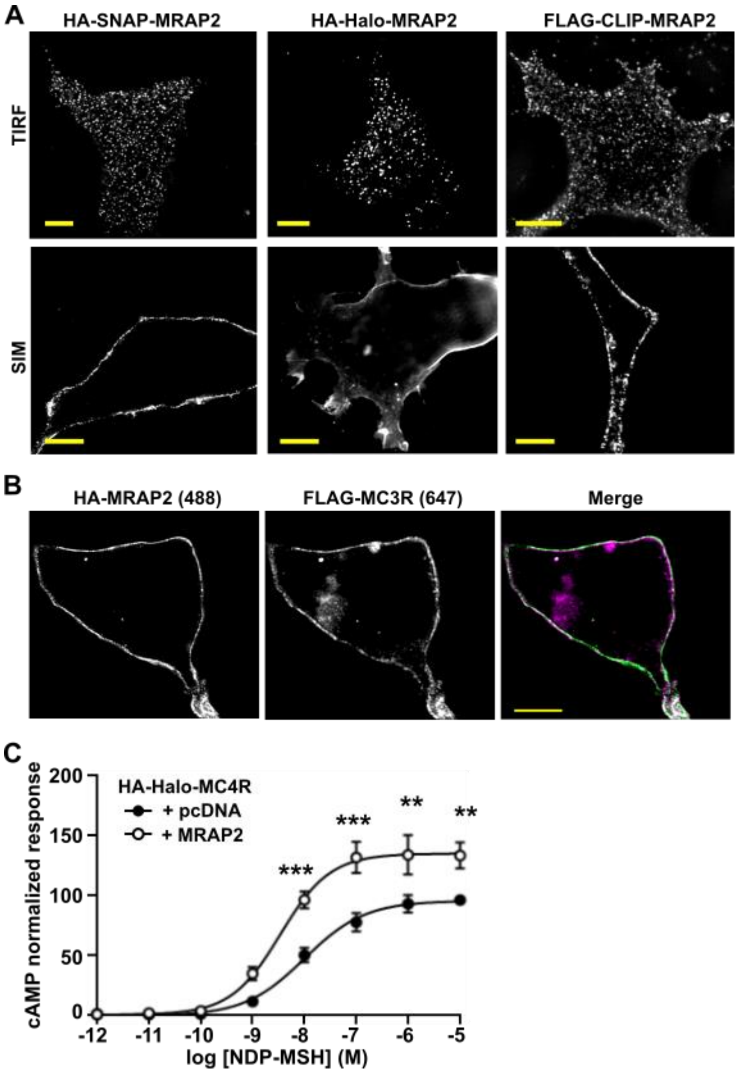

(**A**) Imaging showing expression of the three MRAP2 constructs at the cell surface by TIRF and SIM. Scale, 5 µm. (**B**) SIM imaging showing colocalization of FLAG-CLIP-MC3R and Halo-HA-MRAP2 at the cell surface. Scale, 5 µm. (**C**) MC4R-induced cAMP responses measured by Glosensor in cells transfected with pcDNA or MRAP2, confirming that the MRAP2 plasmids are able to enhance MC4R signaling. AUC was measured and expressed relative to the pcDNA maximal response. N=4. Statistical analyses were performed by two-way ANOVA with Sidak's multiple-comparisons test. ***$p<0.001$, **$p<0.01$.

Figure S4 MC3R and MRAP2 interact in a 1:1 stoichiometry

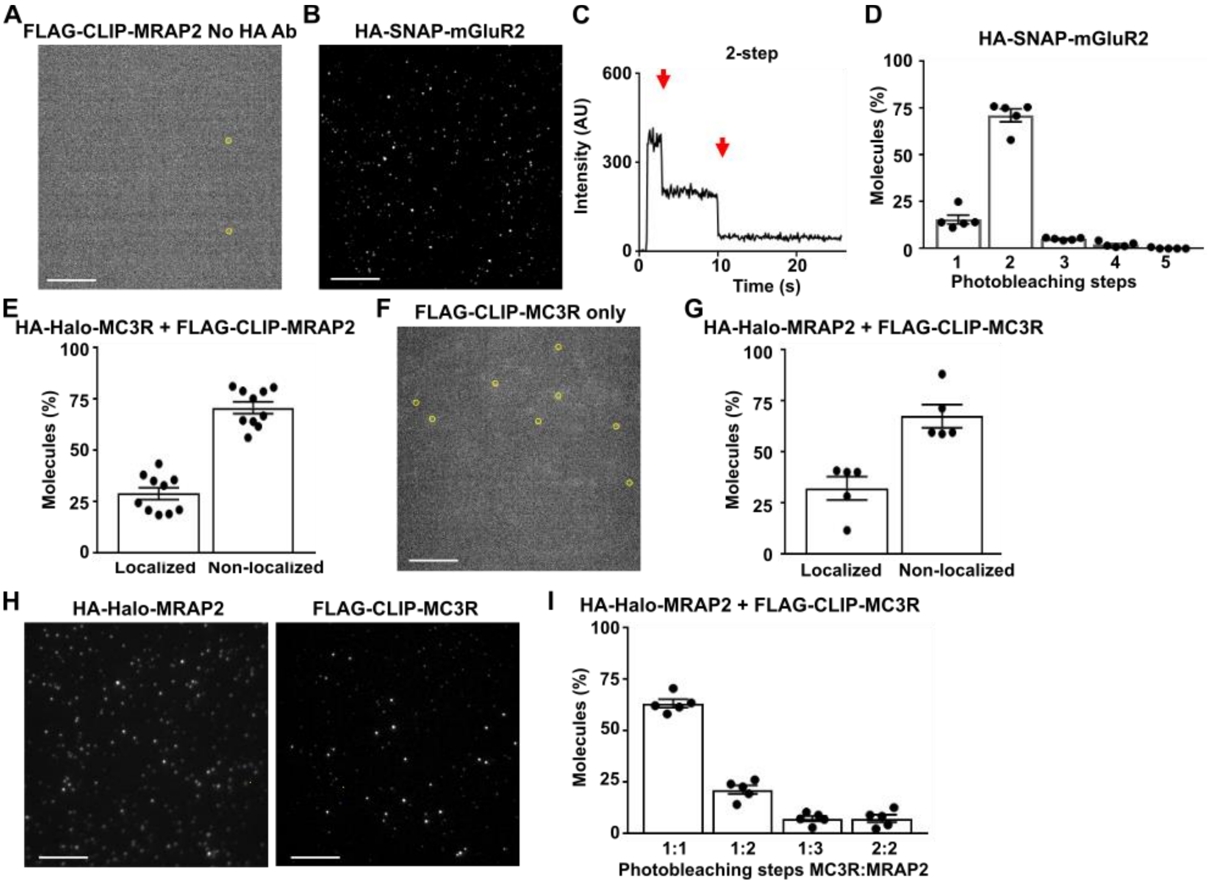

(**A**) Representative image showing little background fluorescence in the absence of anti-HA antibodies. Background fluorescent spots are shown with yellow circles. (**B**) Representative single-molecule fluorescence image of HA-SNAP-mGluR2 with (**C**) examples of single-molecule fluorescence traces with photobleaching steps (red arrows). (**D**) Proportion of molecules with 1 to 5 bleaching steps. N= 1425 molecules from 5 movies. (**E**) Proportion of molecules in two-color SiMPull that are colocalized in cells transfected with HA-Halo-MC3R and FLAG-CLIP-MRAP2. (**F**) Cells transfected with FLAG-CLIP-MC3R only, showing negligible background fluorescence. Background fluorescent spots are shown with yellow circles. (**G**) Proportion of molecules in two-color SiMPull that are colocalized in cells transfected with HA-Halo-MRAP2 and FLAG-CLIP-MC3R. (**H**) Representative two-color SiMPull images of HA-Halo-MRAP2 and FLAG-CLIP-MC3R and (**I**) Photobleaching step analysis from colocalized spots. As in Figure 2, the majority of colocalized spots show 1:1 stoichiometry. N=537 molecules from 5 movies. Scale, 10 μm for all.

Figure S5 MRAP2 does not interact with SSTR3

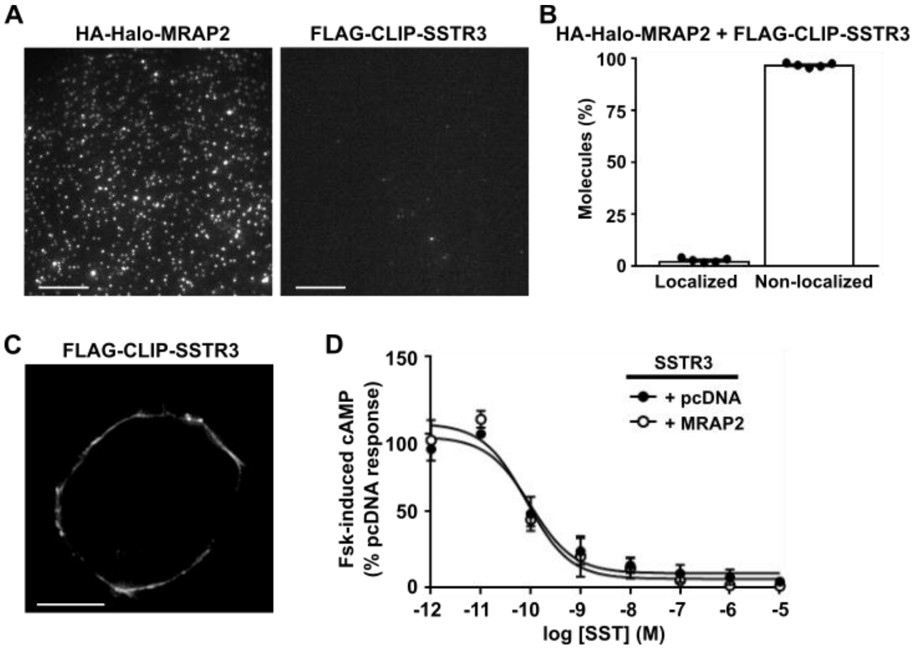

(**A**) Representative two-color SiMPull images of HA-Halo-MRAP2 and FLAG-CLIP-SSTR3, with (**B**) Quantification of the proportion of molecules that are co-localized. N=1790 molecules from 5 movies. Scale, 10 μm. (**C**) SIM image showing FLAG-CLIP-SSTR3 is expressed at the cell surface. Scale, 5 μm. (**D**) FLAG-CLIP-SSTR3-mediated reductions in forskolin (Fsk)-induced cAMP in the presence of pcDNA or MRAP2. N=3.

Figure S6 MRAP2 enhances MC3R activity when equal DNA concentrations are transfected

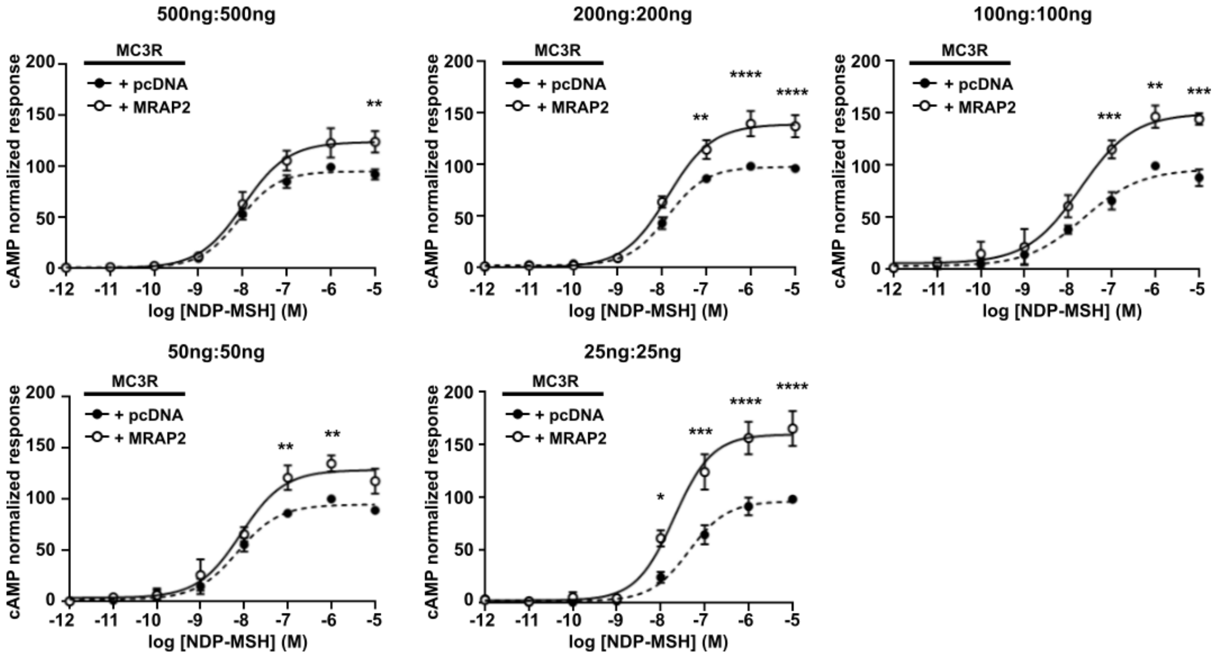

Examination of cAMP responses measured by Glosensor in cells transfected with different total amounts of MC3R with pcDNA or MRAP2. In all assays the same DNA concentration of MC3R and pcDNA or MRAP2 were transfected. AUC was measured and responses expressed relative to the pcDNA maximal response. MRAP2 enhanced MC3R-driven signaling in all assays. N=4. Statistical analyses were performed by two-way ANOVA with Sidak's test. ****$p<0.0001$, ***$p<0.001$, **$p<0.01$. *$p<0.05$.

Figure S7 AlphaFold2 models showing predicted interactions between MC3R and MRAP2

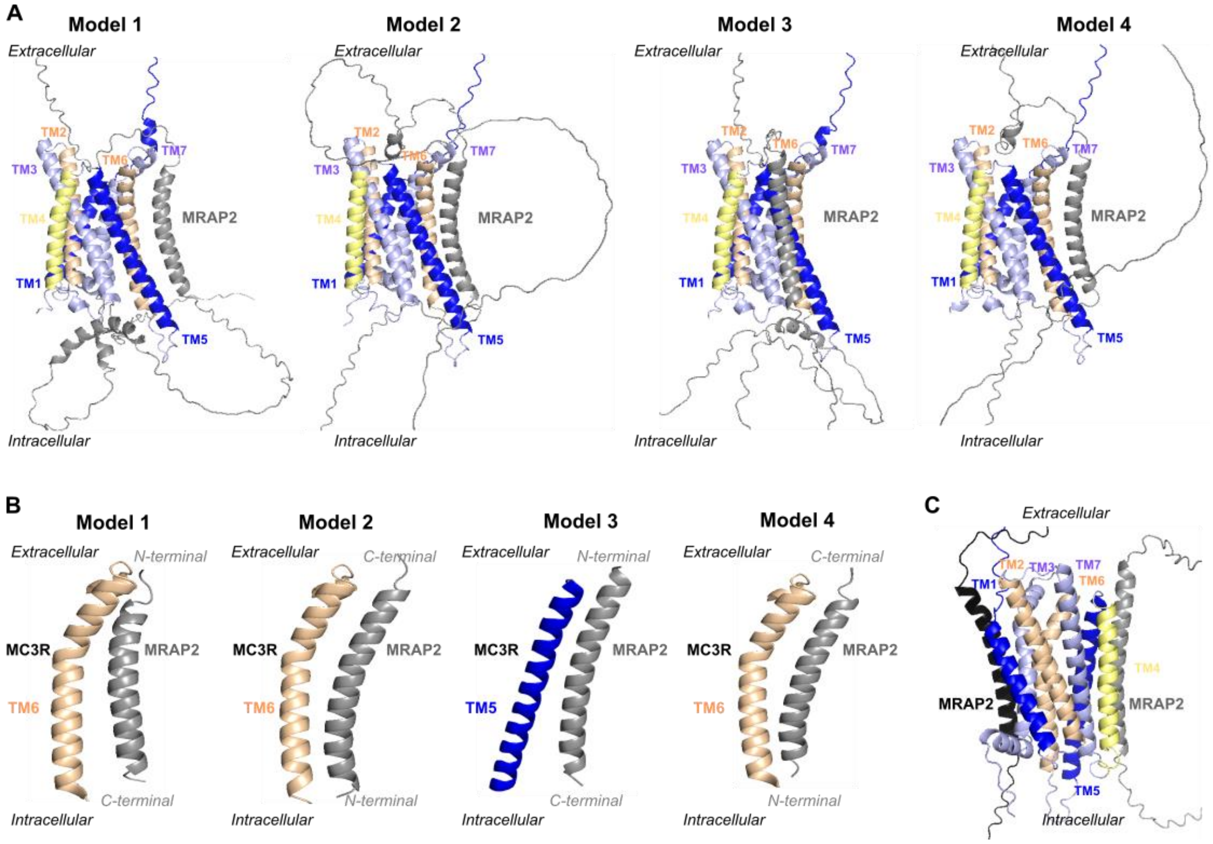

(**A**) Predicted structural models of MC3R and MRAP2 interactions. Model 1 had the highest confidence score, fewer unstructured regions and resembled the MRAP1-MC2R cryo-EM structure (*1*). (**B**) The four models predicted MRAP2 interacts with TM5-TM7, which are known to be important for receptor activation and G protein coupling to MC3R (*2*). Two models predicted MRAP2 inserts into membranes with an extracellular N-terminal region, while two predicted an intracellular N-terminal region. (**C**) Predicted structural model between MC3R and two MRAP2 proteins. All structures similarly predicted the two MRAP2 proteins to bind at distinct sites. No structures predicted MRAP2 dimers.

Figure S8 Effect of MRAP2 alanine mutations on MRAP2 and MC3R expression

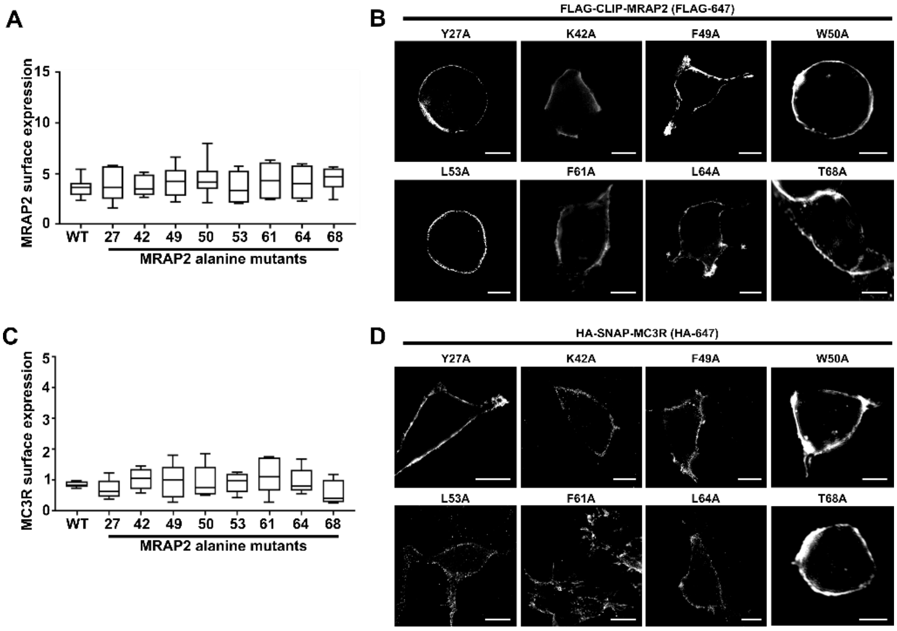

(**A**) Surface expression of MRAP2 assessed in cells transfected with the FLAG-MRAP2 variants. Assays were performed in 96-well plates with primary antibody FLAG and secondary antibody anti-mouse-647. Values were normalized to pcDNA transfected cells. (**B**) MRAP2 surface expression in non-permeabilized cells measured by SIM. Scale, 5 µm. (**C**) Surface expression of MC3R in cells transfected with the HA-HALO-MC3R and FLAG-MRAP2 variants. Assays were performed in 96-well plates with primary antibody HA and secondary antibody anti-mouse-647. Values were normalized to pcDNA transfected cells. (**D**) MC3R surface expression in non-permeabilized cells transfected with the eight alanine variants, measured by SIM. Scale, 5 µm.

Figure S9 Dyngo-4a blocks MC3R internalization

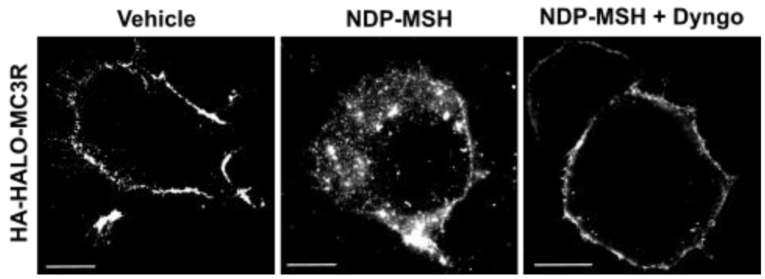

(**A**) Assessment of the effect of Dyngo-4a on cells expressing HA-HALO-MC3R. Cells were exposed to Vehicle, NDP-MSH or agonist with Dyngo-4a and incubated with HA antibody. The HA antibody is only present at the cell surface in vehicle treated cells, while internalization is observed in cells exposed to agonist. In cells pre-treated with Dyngo-4a, there is no internalization observed after 30 minutes exposure to agonist. Scale, 5 μm.

Figure S10 Effect of MRAP2 human variants on MC3R cell surface expression

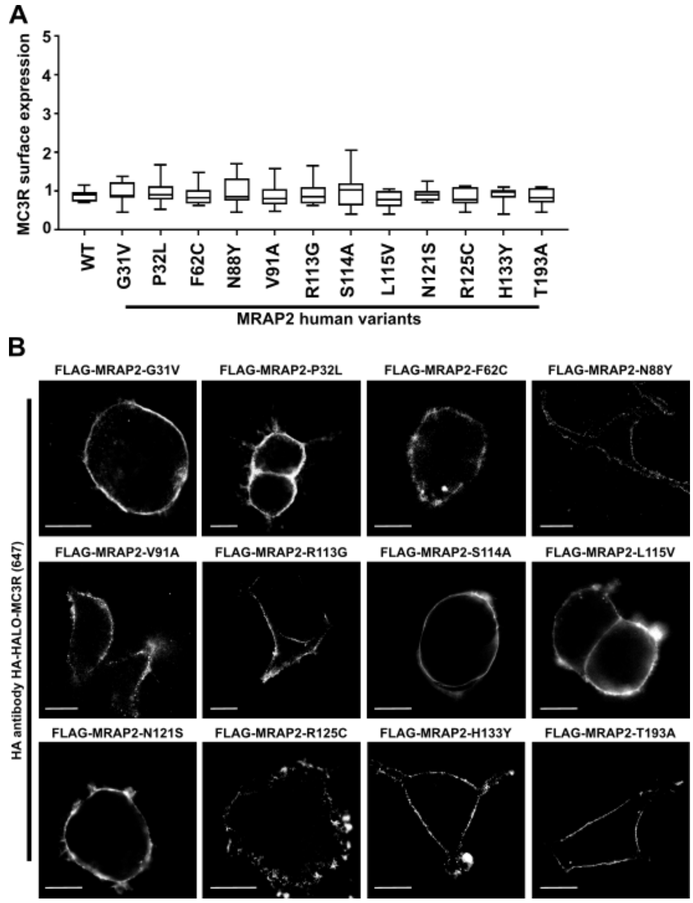

(**A**) Surface expression of MC3R in cells transfected with HA-HALO-MC3R and FLAG-MRAP2 variants. Assays were performed in 96-well plates with primary antibody HA and secondary antibody anti-mouse-647. Values were normalized to pcDNA transfected cells. (**B**) MC3R surface expression in non-permeabilized cells measured by SIM. Scale, 5 μm.

Table S1 Top 15 MC3R-positive and top 15 MC4R-positive clusters in the human HYPOMAP dataset

			% cells/ cluster			Average log normalized expression/ cluster			% co-expressing cells/ cluster	
**Cluster name**	**Predicted region**	**Top marker genes**	**MRAP2**	**MC3R**	**MC4R**	**MRAP2**	**MC3R**	**MC4R**	**MC3R & MRAP2**	**MC4R & MRAP2**
C4-390 Mid-2 GABA-GLU-3 PGR TAC1	ARC	KISS1|NR5A2|SKOR2|PGR	46.94	27.76	20.82	0.41	0.17	0.12	16.73	10.61
C4-388 Mid-2 GABA-GLU-3 PGR CALCR	ARC	PGR|CALCR|NR5A2|LHX4	37.06	23.08	4.9	0.12	0.06	0.01	12.59	2.8
C4-64 Mid-1 GABA-1 NR5A2	Periventricular	SLC6A3|NR5A2|DLK1|MC3R	19.23	21.15	1.92	0.21	0.31	0.03	4.81	0
C4-391 Mid-2 GABA-GLU-3 PGR TAC3	ARC	KISS1|SKOR2|UGT2B7|TAC3	26.82	14.8	15.08	0.21	0.08	0.11	6.7	5.59
C4-161 Mid-1 GABA-6 IL13RA1 GHRH	ARC	GHRH|GAL|ADGRF4|GHSR	8.56	12.83	0	0.07	0.1	0	0.53	0
C4-345 Mid-2 GLU-2 ARHGAP42 COL15A1	VMH	NR5A1|COL15A1|FEZF1|CCBE1	27.8	11.5	12.99	0.23	0.09	0.13	5.32	6.07
C4-375 Mid-2 GABA-GLU-3 POMC ANKRD30A	ARC	SOX3|PGR|POMC|TBX3	69.75	10.92	16.25	0.65	0.04	0.09	8.96	14.01
C4-136 Mid-1 GABA-5 GAL PGR	ARC	GAL|MBNL3|NTS|PGR	24.3	8.41	11.53	0.16	0.06	0.08	1.87	2.49
C4-376 Mid-2 GABA-GLU-3 PDGFD PGR	ARC	PGR|GABRE|ALDH1A1|VGLL3	26.05	7.78	11.08	0.24	0.02	0.06	3.89	5.99
C4-374 Mid-2 GABA-GLU-3 POMC CALCR	ARC	CALCR|POMC|WIF1|PGR	33.69	7.28	2.7	0.27	0.03	0.01	4.31	1.35
C4-349 Mid-2 GLU-2 SLC22A10 CCBE1	VMH	SLC22A10|PGR|DNAH11|ADAMTSL1	50	6.8	9.71	0.45	0.05	0.07	4.37	4.85
C4-385 Mid-2 GABA-GLU-3 PGR DNAH11	ARC	SKOR2|KISS1|DNAH11|VGLL3	22.17	6.6	17.45	0.14	0.02	0.08	2.83	5.66
C4-387 Mid-2 GABA-GLU-3 PGR TRPC6	ARC	PGR|TBX3|PGR-AS1|NR5A2	45.37	6.32	19.19	0.28	0.03	0.11	2.71	10.38
C4-207 Pre-2 GABA-4 SATB2 TNS3	MPOA	SATB2|COL15A1|EGFLAM|ZIC2	39.51	5.85	5.37	0.38	0.03	0.05	4.88	2.44
C4-76 Mid-1 GABA-2 CLMP MBNL3	SCN	NR2F2-AS1|ARHGAP36|SP9|Z96074.1	63.19	5.56	4.17	0.43	0.02	0.03	5.56	2.08
C4-303 Mid-3 GLU-3 SLITRK6 FBN2	MAM	FBN2|TACR3|SIM1|OTP	52.68	0	41.07	0.28	0	0.23	0	25
C4-144 Mid-1 GABA-5 RORB GLI3	MPOA	GLI3|TPTE|GAL|HMX3	58.11	0	31.08	0.49	0	0.23	0	21.62
C4-328 Mid-2 GLU-1 SOX14 CYP19A1	MPOA	SKOR2|CYP19A1|FAM9B|QRFPR	56.45	0	27.96	0.39	0	0.18	0	17.2
C4-194 Pre-2 CHOL-1 BMPR1B	LPOA	SLC5A7|COL6A5|CHAT|LHX8	37.7	0	27.16	0.29	0	0.31	0	13.42
C4-306 Mid-3 GLU-3 CD36 LMCD1	MAM	OTP|KCNH8|LMCD1|STK32B	52.9	0	26.09	0.46	0	0.2	0	15.22
C4-206 Pre-2 GABA-4 LHX6 NR0B1	NA	NPY|SHISAL2B|NR0B1|LHX6	66.23	0	24.68	0.57	0	0.13	0	20.78
C4-396 Mid-2 GLU-4 WNT7B PRRX1	NA	LEF1|WIF1|RSPO3|TRABD2B	18.02	0	22.52	0.12	0	0.15	0	5.41
C4-406 Mid-2 GLU-4 EYA4 NPNT	TMN	WIF1|LEF1|TBX3|VEGFC	33.2	3.86	21.24	0.23	0.01	0.13	2.32	10.81
C4-18 Pre-1 GABA-1 SEMA3C CAV1	NA	CAV1|PROK2|CXCL14|CALCRL	16.67	0	20.6	0.28	0	0.27	0	4.86
C4-171 Pre-2 GABA-1 IL1RAPL2 SST	LPOA	MOXD1|IL1RAPL2|LHX6|NXPH2	62.34	0	20.08	0.81	0	0.21	0	16.32
C4-29 Pre-1 GABA-1 PRKCH PWWP3B	NA	TAC3|SLC22A10|SCML4|EBF1	82.29	0	20	0.77	0	0.1	0	17.14
C4-382 Mid-2 GABA-GLU-3 LEF1 IL1RAPL2	TMN	LEF1|IL1RAPL2|CCN3|ANKRD30A	47.25	0	19.72	0.37	0	0.12	0	11.01
C4-348 Mid-2 GLU-2 SLC22A10 WDR64	VMH	NR2F2-AS1|SLC22A10|SATB1-AS1|CCBE1	44.53	2.73	19.53	0.43	0.03	0.13	0.78	12.11
C4-118 Mid-1 GABA-4 NTS ABCG2	MPOA	TMEM114|ESR1|FAM9B|PGR	78.88	0	19.4	0.7	0	0.08	0	18.53

ARC, arcuate nucleus; MPOA, medial preoptic area; NA, nucleus accumbens; SCN, suprachiasmatic nucleus; TMN, Tuberomammillary nucleus; VMH, ventromedial nucleus of the hypothalamus.

Table S2 Expression plasmids used in this manuscript

Plasmid name	Information	Source
cAMP Glosensor-22F	cAMP sensor	Promega
ss-HA-Halo-MC3R	N-terminal signal peptide from mGluR5, followed by HA, HALO and human MC3R	This manuscript
ss-HA-SNAP-MC3R	N-terminal signal peptide from mGluR5, followed by HA, SNAP and human MC3R	This manuscript
ss-FLAG-CLIP-MC3R	N-terminal signal peptide from mGluR5, followed by HA, CLIP and human MC3R	This manuscript
ss-HA-Halo-MRAP2	N-terminal signal peptide from mGluR5, followed by HA, Halo and human MC3R	This manuscript
ss-HA-SNAP-MRAP2	N-terminal signal peptide from mGluR5, followed by HA, SNAP and human MC3R	This manuscript
ss-FLAG-CLIP-MRAP2	N-terminal signal peptide from mGluR5, followed by HA, CLIP and human MC3R	This manuscript
ss-HA-Halo-mGluR2	Used as template for ss-HA-Halo-MC3R	Joshua Levitz, Weill Cornell Medicine
ss-HA-SNAP-mGluR2	Used as template for ss-HA-SNAP-MC3R	Joshua Levitz, Weill Cornell Medicine
ss-FLAG-CLIP-mGluR2	Used as template for ss-FLAG-CLIP-MC3R	Joshua Levitz, Weill Cornell Medicine
ss-HA-Halo-MC4R	N-terminal signal peptide from mGluR5, followed by HA, HALO and human MC3R	This manuscript
ss-FLAG-CLIP-SSTR3	N-terminal signal peptide from mGluR5, followed by HA, HALO and human SSTR3	This manuscript
SSTR3-Tango	Used as a template for SSTR3 constructs	Bryan Roth, Addgene plasmid #66504
MC3R-Tango	Used as a template for MC3R constructs	Bryan Roth, Addgene plasmid #66429
MRAP2-3xFLAG	Used as a template for MRAP2 constructs and in most assays	Julien Sebag, University of Iowa
B-arrestin2-mYFP	SIM	Robert Lefkowitz, Addgene plasmid #36917
Nluc-Arr2	BRET	Steve Hill, University of Nottingham
Venus-Kras	BRET	Nevin Lambert, Augusta University
Rab5-Venus	SIM	Nevin Lambert, Augusta University
LgC-MC3R	NanoBiT	This manuscript
SmC-MC3R	NanoBiT	This manuscript
LgC-MRAP2	NanoBiT	This manuscript
SmC-MRAP2	NanoBiT	This manuscript
MRAP2-3xFLAG-Y27A	Glosensor, SIM, cell surface expression	This manuscript
MRAP2-3xFLAG-K42A	Glosensor, SIM, cell surface expression	This manuscript
MRAP2-3xFLAG-F49A	Glosensor, SIM, cell surface expression	This manuscript
MRAP2-3xFLAG-W50A	Glosensor, SIM, cell surface expression	This manuscript
MRAP2-3xFLAG-L53A	Glosensor, SIM, cell surface expression	This manuscript
MRAP2-3xFLAG-F61A	Glosensor, SIM, cell surface expression	This manuscript
MRAP2-3xFLAG-L64A	Glosensor, SIM, cell surface expression	This manuscript

MRAP2-3xFLAG-T68A		Glosensor, SIM, cell surface expression		This manuscript
ss-HA-Halo-MC3R-T245A		Glosensor, SIM, cell surface expression		This manuscript
ss-HA-Halo-MC3R-L260A		Glosensor, SIM, cell surface expression		This manuscript
ss-HA-Halo-MC3R-P272A		Glosensor, SIM, cell surface expression		This manuscript
MRAP2-3xFLAG-G31V		Glosensor, SIM, cell surface expression		This manuscript
MRAP2-3xFLAG-P32L		Glosensor, SIM, cell surface expression		This manuscript
MRAP2-3xFLAG-F62C		Glosensor, SIM, cell surface expression		This manuscript
MRAP2-3xFLAG-N88Y		Glosensor, SIM, cell surface expression		This manuscript
MRAP2-3xFLAG-V91A		Glosensor, SIM, cell surface expression		This manuscript
MRAP2-3xFLAG-R113G		Glosensor, SIM, cell surface expression		This manuscript
MRAP2-3xFLAG-S114A		Glosensor, SIM, cell surface expression		This manuscript
MRAP2-3xFLAG-L115V		Glosensor, SIM, cell surface expression		This manuscript
MRAP2-3xFLAG-N121S		Glosensor, SIM, cell surface expression		This manuscript
MRAP2-3xFLAG-R125C		Glosensor, SIM, cell surface expression		This manuscript
MRAP2-3xFLAG-H133Y		Glosensor, SIM, cell surface expression		This manuscript
MRAP2-3xFLAG-T193A		Glosensor, SIM, cell surface expression		This manuscript

Table S3 Identification of possible contacts between MRAP2 and MC3R in AlphaFold2 models

		MC3R				
**MRAP2**	**Region[a]**	**Rank 1**	**Rank 2**	**Rank 3**	**Rank 4**	**No. of models[b]**
**W23**	EC/IC		N68			1
**E26**	EC/IC	D117				1
**Y27**	EC/IC	F281	K239, G240	D121	Y299, R302	**4**
**I30**	EC/IC		Q233, H235		S236	2
**K39**	EC/IC	F34				1
**A40**	EC/IC	Y273	M238			2
**K42[c]**	TM	P272, Y273		M187		2
**Y43**	TM		V242		M238	2
**S44**	TM		F211			1
**I45**	TM			M187		1
**V46**	TM			V190	T245	2
**F49**	TM	L260		C191, M195	G249	**3**
**W50[c]**	TM	P257	T245, L248, G249		L248	**3**
**L53[c]**	TM	A256		A198	I252	**3**
**F61[c]**	TM		V263	T205	F197	**3**
**L64[c]**	TM	T245	L260	L206	L260	**4**
**L67**	TM	F211, R215				1
**T68**	TM		Y267	H209		**2**
**F90**	IC/EC		D117			1
**R113**	IC/EC	P225				1
**I120**	IC/EC			Q223		1
**F169**	IC/EC	S236				1

The Rank 1 model has fewer unstructured regions and is most similar to the published MRAP1-MC2R structure (*1*). [a]Region refers to the structural location as either: EC, extracellular region, TM, transmembrane helix, IC, intracellular region. Some residues are designated as IC/EC as the orientation of MRAP2 differs in some models (Figure S7). [b]No. of models refers to the total number (out of 4) of structural models that identified a link between the MRAP2 residue and MC3R. MRAP2 residues highlighted in red were mutated to alanine and functionally characterized in this study, while MC3R residues similarly investigated are highlighted in blue. [c]Alanine substitutions that impair MRAP2 enhancement of MC3R signaling (Figure 5).

Table S4 **Densitometry of FLAG-MRAP2 protein with alanine variants**

**Human MRAP2 (relative to WT)**	**Mean±SEM (N)**
Y27A	1.13 ± 0.03 (5)
K42A	1.04 ± 0.06 (5)
F49A	0.97 ± 0.06 (5)
W50A	1.11 ± 0.03 (5)
L53A	0.93 ± 0.07 (5)
F61A	1.04 ± 0.09 (5)
L64A	0.93 ± 0.09 (5)
T68A	1.68 ± 0.19 (5)****
pcDNA	0.01 ± 0.01 (5)****

Densitometry of FLAG-MRAP2 protein from five western blots. MRAP2 variants were investigated in separate batches and protein expression was normalized to the MRAP2-WT control in each experiment. Asterisks indicate significant difference to WT. ****$p<0.0001$ compared to MRAP2-WT.

Table S5 Identification of possible contacts between MC3R and MRAP2 residues mutated in overweight and/or obese individuals

Variant	Rank 1	Rank 2	Rank 3	Rank 4
**G31V**	E29	None	V33	None
**P32L**	S34	None	S34	None
**F62C**	None	I58, L66	None	None
**N88Y**	None<br>Mutant forms new contact with S89.	R84, S92. Mutant forms new contacts with F85, R86.	None	S92<br>Mutant forms new contact with R84.
**V91A**	None	None	None	F94
**R113G**	Link to MC3R P225.<br>Mutant loses contact.	None	E111	None
**S114A**	Link to MC3R N159.<br>Mutant loses contact.	None	None	None
**L115V**	None	None	None	None
**N121S**	H117, C118, R125, N171	None	H117, C118, R125, E124.<br>Mutant loses H117 contact.	None
**R125C**	N121, A129	None	N121, E122	None
**H133Y**	K130	None	K130<br>Mutant loses contact.	None
**T193A**	None	None	None	None

Contacts are in MRAP2 (i.e. intramolecular) unless otherwise stated. The predicted consequences of MRAP2 variants are highlighted in red. Those in which there is no red text predicted no structural effect of the MRAP2 variant.

References

1. P. Luo, W. Feng, S. Ma, A. Dai, K. Wu, X. Chen, Q. Yuan, X. Cai, D. Yang, M. W. Wang, H. Eric Xu, Y. Jiang, Structural basis of signaling regulation of the human melanocortin-2 receptor by MRAP1. *Cell Res* **33**, 46-54 (2023).
2. W. Feng, Q. Zhou, X. Chen, A. Dai, X. Cai, X. Liu, F. Zhao, Y. Chen, C. Ye, Y. Xu, Z. Cong, H. Li, S. Lin, D. Yang, M. W. Wang, Structural insights into ligand recognition and subtype selectivity of the human melanocortin-3 and melanocortin-5 receptors. *Cell Discov* **9**, 81 (2023).
